# Study on Wavelet Packet Energy Characteristics on Friction Signal of Lapping with the Fixed Abrasive Pad

**DOI:** 10.3390/mi13070981

**Published:** 2022-06-21

**Authors:** Zhankui Wang, Zhao Zhang, Shiwei Wang, Minghua Pang, Lijie Ma, Jianxiu Su

**Affiliations:** 1 Postdoctoral Research Base, Henan Institute of Science and Technology, Xinxiang 453003, China; zhangzhao_zz0309@126.com (Z.Z.); wangshiwei_1208@163.com (S.W.); pangminghua909@163.com (M.P.); mlj001@163.com (L.M.); dlutsu2004@126.com (J.S.); 2 Postdoctoral Station, Henan University of Science and Technology, Luoyang 471000, China

**Keywords:** fixed abrasive pad, friction and wear, quartz glass, material removal rate, surface quality, wavelet packet energy coefficient

## Abstract

The surface condition of the fixed abrasive pad (FAP) has a significant impact on its machining performance, workpiece material removal rate (MRR), and surface roughness. To clarify the wavelet packet energy characteristics of friction signal under different surface conditions of FAP and its mapping relationship with MRR and workpiece surface quality, FAP samples in different processing stages were obtained through a consolidated abrasive grinding quartz glass experiment. Then, the friction signals in different stages were received by the friction and wear experiment between the FAP and quartz glass workpiece, and the wavelet packet analysis was carried out. The experimental results show that with the increase of lapping time, the surface wear degree of the FAP increased gradually, and the MRR of the workpiece, the surface roughness of the FAP, and the surface roughness of the workpiece decreased slowly. In the wavelet packet energy of friction signal during machining, the energy proportion of frequency band 7 showed an upward trend with the increase of lapping time. The energy proportion of frequency band 8 showed a downward trend with the increase of lapping time. The change characteristics of the two are significantly correlated with the surface condition of the FAP.

## 1. Introduction

With the development of science and technology, people put forward higher requirements for the quality control and machining efficiency of ultra-precision machining technology [1]. The traditional free abrasive machining technology has the following disadvantages: uncontrolled abrasive trajectory, low abrasive utilization, high production cost, environmental pollution, and so on [2,3,4,5]. To overcome the shortcomings of free abrasive, the 3M company first proposed the consolidated abrasive polishing technology, which consolidates the abrasive on the cushion substrate and transforms the three-body removal of workpiece material into two-body removal, which is easier to control [6,7].

In consolidated abrasive polishing, the surface material of the workpiece is mainly removed by mechanical and chemical actions such as friction and extrusion between abrasive particles and the workpiece. The friction signal between its interface reflects the real-time tribological characteristics of the polishing pad surface [8], and it is a non-stationary signal, which is difficult to be analyzed by traditional Fourier transform. Therefore, when analyzing the friction signal, it is necessary to use the analysis methods of non-stationary signal, such as wavelet transform, short-time Fourier transform, Hilbert Huang transform, etc. [9,10,11]. At present, many scholars have studied the analysis of non-stationary signals. Xiong et al. [12] proposed a novel bearing fault diagnosis method based on the Wavelet Packet Transform (WPT) and a lightweight variant of Deep Residual Networks (DRN). The accuracy of fault identification is high, which has great potential in the practical application of industrial fault diagnosis. Habbouche et al. [13] proposed a new data-driven approach for bearing prognostics based on the decomposition of wavelet packets and bidirectional long short-term memory for preprocessing and tracking degradation. Han et al. [14] proposed the modal parameter identification method and damage diagnosis method based on Hilbert Huang Transform (HHT) to monitor the bridge structure. Lan et al. [15] used short-time Fourier transform to analyze the vibration signal and explored structural vibration’s time-varying characteristics and modal frequency characteristics. Xu et al. [16] proposed a rolling bearing fault detection method based on Translation-Invariant Denoising (TID) and HHT, which can effectively extract the fault features of bearings. Li et al. [17] realized the feature extraction of weak friction vibration signal through harmonic wavelet packet transform. J. Rabi et al. [18] proposed a method based on wavelet transform and empirical mode decomposition to analyze the defect vibration signal of friction stir welding tunnel. Yao et al. [19] proposed a rolling bearing fault diagnosis method based on wavelet packet analysis and a limit learning machine, which can accurately diagnose the bearing fault category.

To sum up, there have been many research reports on the analysis and processing of non-stationary signals, but how to analyze the friction in the process of lapping and find the characteristics of friction signal reflecting the surface condition of the FAP has not been studied. Therefore, this paper proposes to use the wavelet packet analysis method to analyze the mechanical friction signal in the processing of the FAP by comparing the proportion of wavelet packet energy in each frequency band of friction force signal under different FAP surface conditions to obtain the friction signal characteristics reflecting the surface condition of the FAP.

## 2. Basic Principles

**Figure 1:**
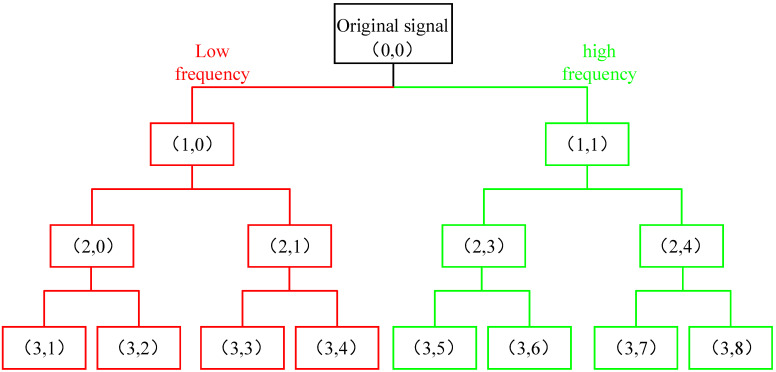
Decomposition diagram of three-layer wavelet packet.

Wavelet packet analysis is a new method based on wavelet analysis [20] which can decompose the high-frequency and low-frequency parts of the signal more finely and conduct a more comprehensive signal analysis [21]. Each wavelet packet decomposition will obtain two sub-bands of low frequency and high frequency. The three-layer wavelet packet decomposition is shown in Figure 1. For the n-layer decomposed wavelet packet signal, 2n sub-bands are obtained. The decomposition calculation formula is as follows:

(1) $$\;\;\;d_{i,j,2m}=\underset{k}{\sum }h\left(k-2i\right)d_{k,j+1,m}$$

$$d_{i,j,2m+1}=\underset{k}{\sum }g\left(k-2i\right)d_{k,j+1,m}$$

The calculation formula of wavelet packet reconstruction is as follows:

(2) $$d_{i,j+1,m}=\underset{k}{\sum }h\left(i-2k\right)d_{i,j,2m}+\underset{k}{\sum }g\left(i-2k\right)d_{i,j,2m+1}$$

where, $d_{i,j,m}$ is the *i*-th wavelet packet coefficient of the *m*-th node of layer *j*; and *h*(*k*) and *g*(*k*) are low-pass and high pass filter coefficients, respectively.

The principle of wavelet packet energy is to solve the signal energy on different decomposition scales and arrange these energy values into eigenvectors according to the scale order for recognition [22,23]. Wavelet packet energy contains rich signal characteristics, and its wavelet packet decomposition result is $d_{i,j}\left(k\right)$ energy *E* in different frequency bands $E_{i,j}$. The calculation formula is:

(3) $$E_{i,j}=\overset{N}{\underset{k=1}{\sum }}{\left|d_{i,j}\left(k\right)\right|}^{2},j=0,1,\cdot \cdot \cdot 2^{i}-1$$

where *N* is the original signal length. All $E_{i,j}$ constitute the wavelet packet energy spectrum. The proportion of wavelet packet energy $P_{i,j}$, the calculation formula is as follows:

(4) $$P_{i,j}=\frac{E_{i,j}}{\sum_{j=0}^{2^{i}-1}E_{i,j}}$$

## 3. Experimental Part

### 3.1. Experimental Design

**Figure 2:**
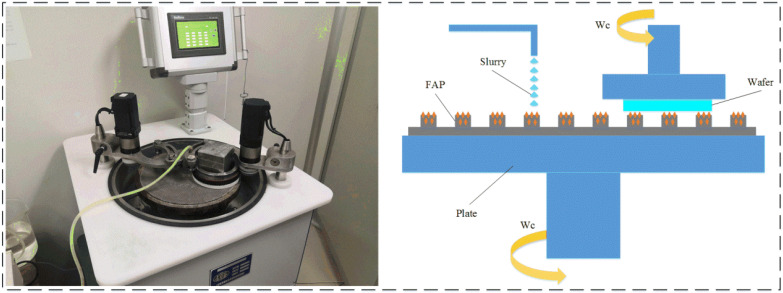
Ultra-precision plane lapping and polishing machine and its processing principle.

**Figure 3:**
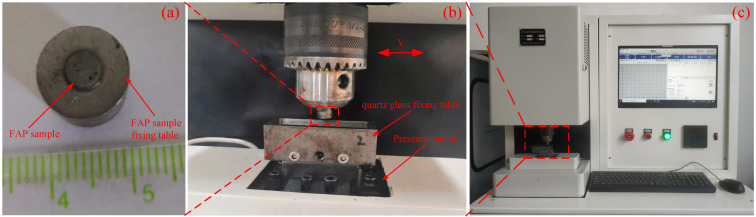
The experimental equipment and partial enlarged view. Note: (**a**) FAP sample; (**b**) local amplification; (**c**) experimental equipment.

**Figure 4:**
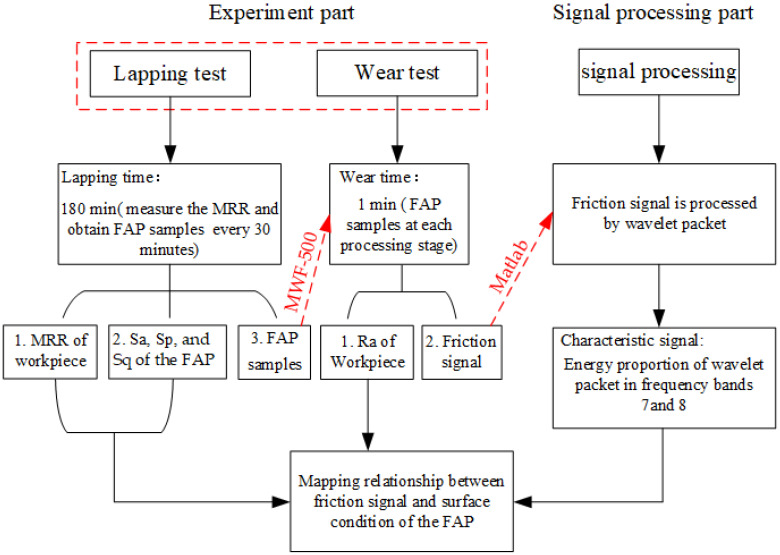
Flow chart for experiment.

The test part mainly includes the lapping test and the wear test. The lapping test is carried out on the ZDHP-30B ultra precision plane polishing machine. The lapping pad is W7 fixed abrasive pad, and the lapping workpiece is a quartz glass wafer with a diameter of 50 mm and a thickness of 3 mm. Its initial surface roughness is 60–70 nm. The total lapping time is 180 min. Every 30 min in the lapping process is a stage. After each stage, the quality difference of the workpiece is measured to calculate the MRR of the processing stage. The processing performance of the FAP is characterized by the material removal rate. FAP samples are obtained from the surface of the polishing pad for standby to observe the surface wear condition of FAP samples. The FAP does not need to be dressed. The next stage of lapping quartz glass is carried out, and so on until the grinding time reaches 180 min. The lapping test processing equipment and processing principle are shown in Figure 2. Other process parameters are shown in Table 1. The wear test is performed after each stage of the lapping test. The wear test was carried out on a microcomputer controlled reciprocating friction and wear tester (MWF-500). Its working principle is that the spindle rotates to drive the slider crank mechanism to move, so as to realize reciprocating linear motion. Paste the FAP samples of each processing stage obtained from the lapping test on the cylindrical workbench. The FAP samples lap the quartz glass in a straight-line reciprocating motion on the reciprocating friction and wear tester with a reciprocating distance of 6 mm. Thus, the friction signal generated in the process of FAP lapping quartz glass can be obtained. The test equipment and FAP sample diagram are shown in Figure 3: 150 r/min refers to the rotation speed of the spindle of MWF-500 in the wear test; the unit of the instrument is r/min; 100 rmp represents the rotation speed of the polishing disc of ZDHP-30B in the lapping test. The unit of the instrument is rpm. The effect of the two instruments is consistent at specific speeds. When the spindle speed of the wear test is 150 r/min, the effect is consistent with that of the polishing disc speed of 100 rmp in the lapping test. Other parameters are shown in Table 2. The experimental flow chart is shown in Figure 4.

### 3.2. Measurement and Characterization

#### 3.2.1. Material Removal Rate

In each lapping experiment, SatoriousCP225D precision balance is used to weigh the mass of quartz glass samples before and after processing. The diameter of quartz glass is measured with a micrometer. Finally, the weight loss method calculates the MRR of the quartz glass in the lapping process.

(5) $$\mathrm{MRR}=\frac{\Delta m}{\rho \times s\times t}\times 10^{7}$$

where “MRR” is the material removal rate, nm/min; Δm is the poor quality of quartz glass before and after processing, g; ρ is the density of quartz glass, ρ = 2.2 g/cm^3^; s is the workpiece area, cm^2^; and t is the processing time, min.

#### 3.2.2. Observation of Surface Roughness and Three-Dimensional Morphology

Before and after the lapping test, Contour GT-X3/X8 white light interferometer was used to measure the surface roughness Sa and three-dimensional surface morphology of the FAP. Before and after the wear test, Contour GT-X3/X8 white light interferometer was used to measure the surface roughness Sa of the workpiece. When the measuring point was selected in the experiment, we chose 3 points on a straight line on the surface of the polished workpiece as measurement points, which can better reflect the surface roughness Sa and three-dimensional topography of the polished workpiece, and the average value was taken as the final measurement result.

## 4. Results and Discussion

### 4.1. Relationship between the MRR and Energy Proportion of Wavelet Packet 

**Figure 5:**
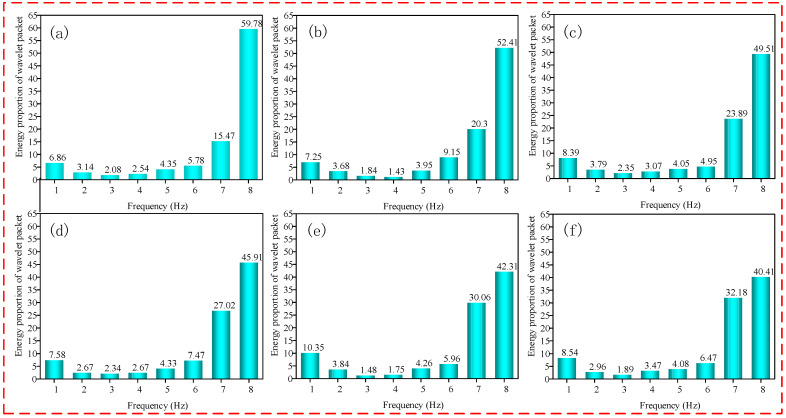
Proportion of wavelet packet energy in each processing stage of the FAP. Note: (**a**) Lapping time 0–30 min; (**b**) lapping time 30–60 min; (**c**) lapping time 60–90 min; (**d**) lapping time 90–120 min; (**e**) lapping time 120–150 min; (**f**) lapping time 150–180 min.

**Figure 6:**
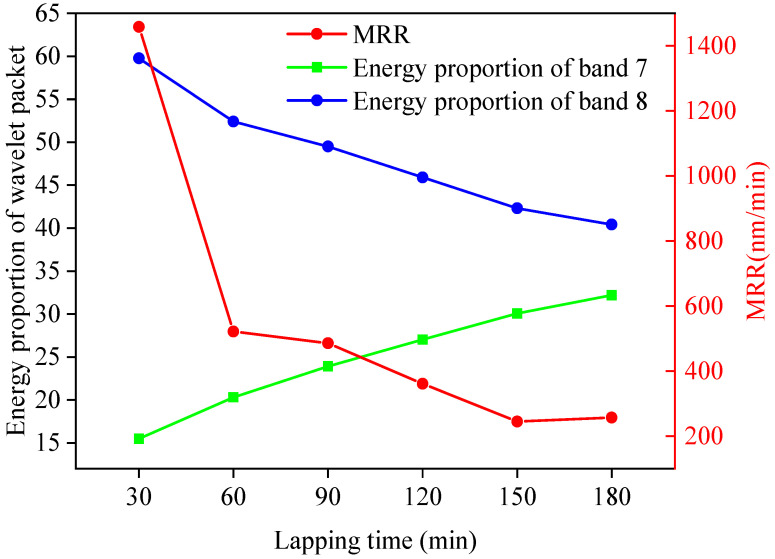
Relationship between the MRR and energy proportion of frequency bands 7 and 8.

MATLAB software was used to process the friction signal, and the db2 wavelet function was selected; the length of the signal was N = 600, and the theoretical maximum number of decomposition layers was 4. After three-layer wavelet packet decomposition, 8 frequency bands from low to high were obtained to meet the subdivision of the signal and reduce the amount of calculation. Finally, three-layer wavelet packet decomposition was selected. When the decomposition scale was 3, the corresponding frequency bands of the 8 signal nodes are shown in Table 3. The distribution of energy in different frequency bands was calculated, and the proportion of wavelet packet energy in each processing stage was obtained, as shown in Figure 5. It can be seen from Figure 5 that the energy was mainly concentrated in frequency bands 7 and 8, and the energy of the low-frequency part accounted for a relatively low proportion. Therefore, the fault signal mainly existed in the high-frequency part. The relationship between the energy proportion in frequency bands 7 and 8 and the MRR was obtained by associating frequency band 7 and 8 with the MRR, as shown in Figure 6. 

### 4.2. Relationship between Surface Roughness of the FAP and Energy Proportion of Wavelet Packet 

**Figure 7:**
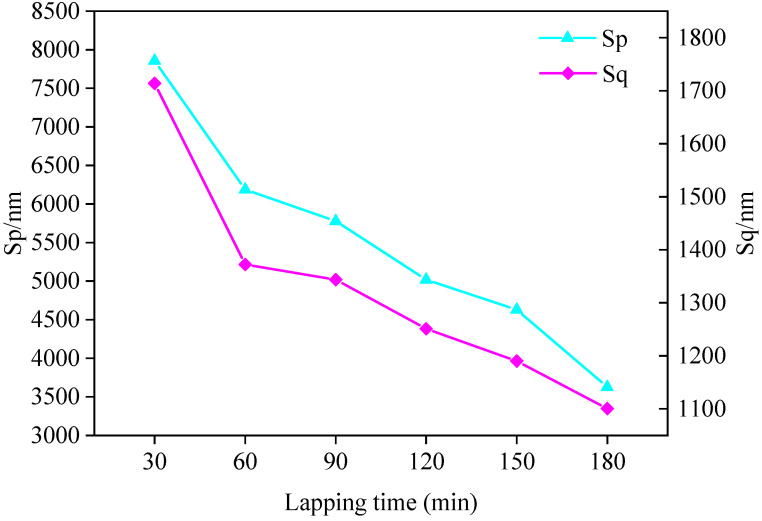
The three-dimensional surface parameters Sp and Sq of FAP at each processing stage.

**Figure 8:**
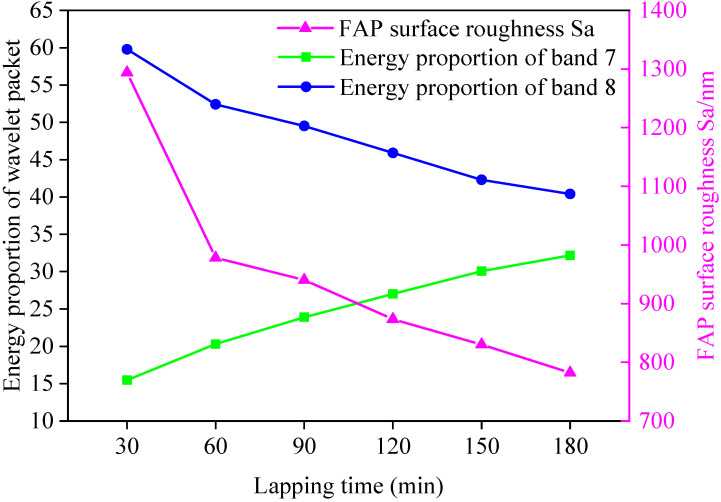
Relationship between surface roughness of the FAP and energy proportion of frequency bands 7 and 8.

**Figure 9:**
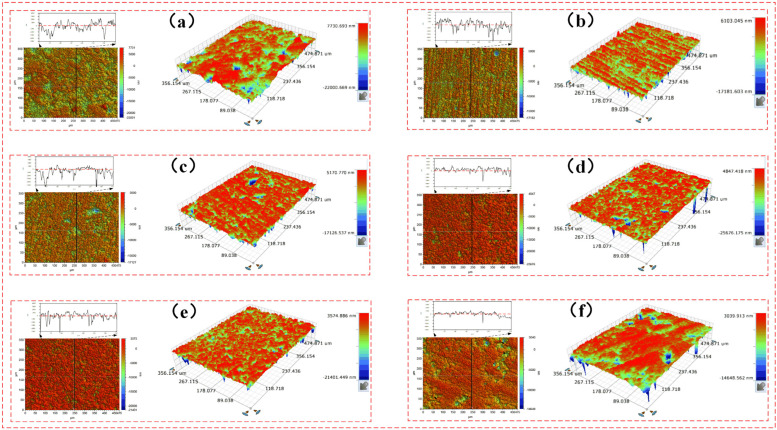
Three-dimensional surface morphology of the FAP in different processing stages. Note: (**a**) Lapping time 0–30 min; (**b**) lapping time 30–60 min; (**c**) lapping time 60–90 min; (**d**) lapping time 90–120 min; (**e**) lapping time 120–150 min; (**f**) lapping time 150–180 min.

The surface roughness and three-dimensional surface parameters of the FAP after each processing were measured. It can be seen from the literature that the size of peak SP can characterize the protruding height of surface abrasive particles, and the size of arithmetic square root Sq can characterize the condition of surface texture [24]. The surface roughness and three-dimensional surface parameters of the FAP in each processing stage are shown in Table 4. The three-dimensional surface parameters Sp and Sq of FAP at each processing stage are shown in Figure 7. The relationship between the surface roughness of the FAP in each processing stage and the energy proportion of frequency bands 7 and 8 are as shown in Figure 8. The three-dimensional morphology of its surface is shown in Figure 9. It can be seen from Figure 7 that Sp and Sq gradually decreased with the increase of lapping time because the FAP surface abrasive particles were constantly worn during the lapping process, resulting in the continuous reduction of the abrasive particle height on the FAP surface. It can be seen from Figure 8 that the changing trend of energy proportion in frequency band 8 of wavelet packet was consistent with the changing trend of surface roughness of the FAP, which decreased with the increase of lapping time. The energy proportion in frequency band 7 of the wavelet packet was opposite to the changing trend of surface roughness of the FAP and increased with the increase of lapping time. This is because, at the initial stage of lapping, there were many exposed abrasive particles on the FAP surface, and the exposed height of abrasive particles is high. The edge tip of the abrasive particles was complete, the micro-cutting ability of the abrasive particles was strong, and the surface roughness was large. During the lapping process, a large number of abrasive particles scratched the surface of the workpiece. The energy of the wavelet packet in frequency band 8 was large, and that in the frequency band 7 was small. With the increase in lapping time, the abrasive particles on the surface of the FAP aggravated the wear and fall off. More and more matrix materials were exposed on the surface, and their SP and Sq decreased. FAP surface roughness was small, which affected the micro-cutting ability of the abrasive particles to a certain extent. A large number of substrates contacted and scratched the workpiece, which increased the energy of frequency band 7 and decreased the energy of frequency band 8 of the wavelet packet.

### 4.3. Relationship between Surface Roughness of Workpiece after Wear and the Energy Proportion of Wavelet Packet

**Figure 10:**
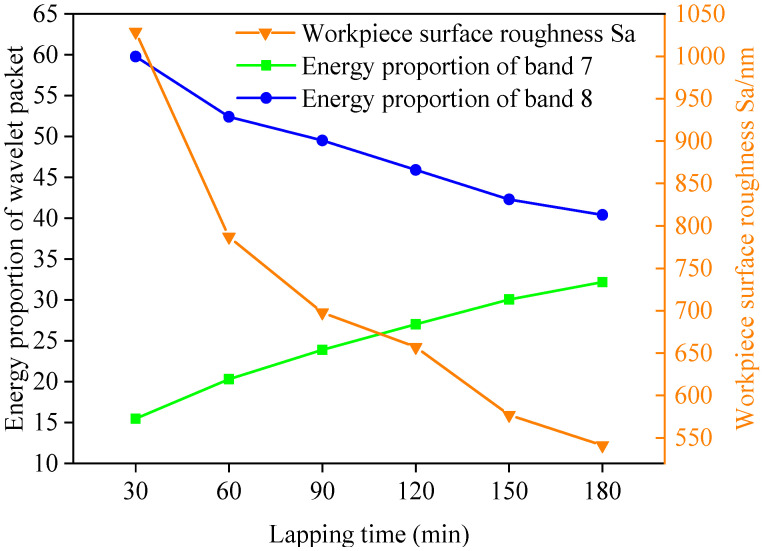
Relationship between workpiece surface roughness and energy proportion of frequency bands 7 and 8.

**Figure 11:**
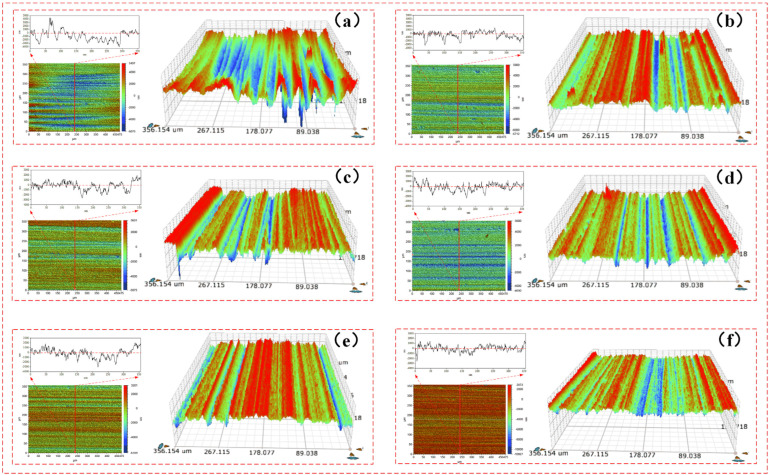
The three-dimensional morphology of quartz glass workpiece lapping by FAP samples at different processing stages. Note: (**a**) The FAP samples (0–30 min) lapping quartz glass workpiece; (**b**)the FAP samples (30–60 min) lapping quartz glass workpiece; (**c**) the FAP samples (60–90 min) lapping quartz glass workpiece; (**d**) the FAP samples (90–120 min) lapping quartz glass workpiece; (**e**) the FAP samples (120–150 min) lapping quartz glass workpiece; (**f**) the FAP samples (150–180 min) lapping quartz glass workpiece.

In order to observe the micro cutting performance of FAP surface abrasive particles and the surface roughness of workpiece at different machining stages more intuitively, the FAP samples were ground back and forth on a microcomputer controlled reciprocating friction and wear tester (MWF-500). The surface roughness and three-dimensional morphology of lapping quartz glass of FAP samples were measured at different processing stages after each wear test. The surface roughness of lapping quartz glass of FAP samples at each processing stage is shown in Table 5. The relationship between the surface roughness of quartz glass and the energy proportion of frequency bands 7 and 8 is shown in Figure 10. The three-dimensional morphology of quartz glass workpiece lapping by FAP samples at different processing stages is shown in Figure 11. It can be seen from Figure 10 that the changing trend of energy proportion in frequency band 8 of wavelet packet was consistent with the changing trend of surface roughness of quartz glass workpiece, which decreased with the increase of lapping time. The energy proportion of frequency band 7 was the opposite of the changing trend of the surface roughness of quartz glass workpiece and increased with the increase of lapping time. This is because many abrasive particles were exposed to the FAP surface at the initial stage of lapping, and the height was high. The abrasive particles scratched the workpiece surface during the lapping process and left large scratches. The workpiece surface roughness was large, and the abrasive particles had a strong micro-cutting performance. The energy proportion of frequency band 8 was large, and the energy proportion of frequency band 7 was small. The abrasive particles wore and fell off with the increased lapping time, and many matrices were exposed and scratched on the workpiece surface. The damage to the workpiece surface was small, the surface roughness was reduced, and the micro-cutting ability of abrasive particles was weakened. The energy proportion of frequency band 7 was large, and the energy proportion of frequency band 8 was small.

### 4.4. Discussion

It can be seen from the above that the MRR of the workpiece was consistent with the change law of the surface roughness of the FAP and the surface roughness of the workpiece, which generally showed a downward trend with the increase of lapping time. The energy proportion in frequency bands 7 and 8 of the wavelet packet was relatively high. The energy proportion in the frequency band 7 increased with the increase of lapping time, and the energy proportion in the frequency band 8 decreased with the increase of lapping time.

**Figure 12:**
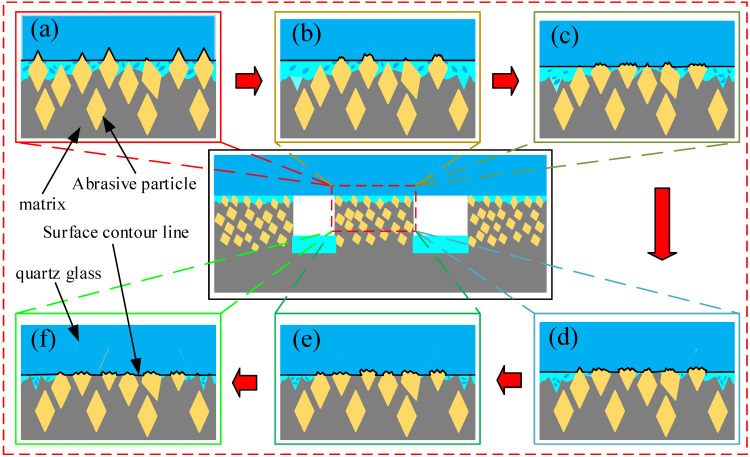
Machining wear principle of the FAP. Note: (**a**) Lapping time 0–30 min; (**b**) lapping time 30–60 min; (**c**) lapping time 60–90 min; (**d**) lapping time 90–120 min; (**e**) lapping time 120–150 min; (**f**) lapping time 150–180 min.

The FAP was mainly composed of diamond abrasive particles and resin matrix. It can be seen that there are two reasons for the change of energy proportion in frequency bands 7 and 8. On the one hand, there were a large number of diamond abrasive particles on the surface of the FAP. The diamond abrasive particles achieved a certain MRR by scratching and ploughing the workpiece surface in the processing process. At the initial stage of lapping, the wear degree of the abrasive particles on the surface of the FAP was small, most of the abrasive particles retained the complete blade tip, the surface morphology was relatively convex, and the surface roughness was large, as shown in Figure 9a. Its processing performance was not greatly affected. The abrasive tip cut deep into the workpiece surface, scratched the plough workpiece’s surface, and left deep scratches. There were many waste chips generated by scratching, and its MRR was large, as shown in Figure 12a. In machining, the scrap and abrasive particles contact together and scratch the workpiece surface, resulting in the change of wavelet packet energy. Therefore, the energy of frequency band 8 was the largest in the initial lapping stage. With the increase in lapping time, the abrasive particles on the surface of the FAP were constantly worn, part of the abrasive particles were forced to fall off from the surface of the FAP, and the edge tips of the other abrasive particles had a large area of edge collapse. The abrasive protrusion on the surface of the FAP decreased, the surface became relatively smooth, and the surface roughness decreased. The cutting performance of worn abrasive particles was greatly reduced. In machining, the depth of abrasive particles cutting into the surface of the workpiece became shallow, and the amount generated debris became smaller. The times of scraping the surface of the workpiece with debris and abrasive particles were reduced, and the energy proportion in frequency band 8 were reduced.

On the other hand, the FAP was mainly composed of a resin matrix, and the abrasive particles were fixed on the matrix. At the initial stage of lapping, the abrasive particles on the surface of the FAP were only slightly worn, and the abrasive particles were more prominent. The abrasive particles scratched the cutting workpiece to remove the material in the lapping process. Only a small part of the matrix with high protrusion contacted the workpiece surface. The energy change caused by this part of the matrix scratching the workpiece surface was small, so the energy proportion of frequency band 7 was small in the initial lapping stage. As the lapping continued, the abrasive particles on the surface of the FAP accelerated the wear, a large number of abrasive particles fell off or collapsed on the surface, the height of abrasive particles decreased, and more matrices were exposed and participated in scratching the workpiece surface, as shown in Figure 12e. The energy change caused by the matrix scratching the workpiece surface increased, and the energy proportion in the frequency band 7 increased. Due to the large difference in hardness between the matrix and diamond abrasive particles, the scratches left by the matrix scratching the workpiece surface were shallow and dense. The workpiece surface morphology was relatively smooth and had small surface roughness, as shown in Figure 11f. This is consistent with the experimental results in Section 4.3.

## 5. Conclusions

With the increase of lapping time, the MRR of the workpiece, the surface roughness of the FAP, and the surface roughness of the workpiece after wear were consistent, and all showed a downward trend with the increase of lapping time.

The wavelet packet energy was mainly concentrated in the high-frequency part of the lapping process. The energy proportion in the frequency band 7 increased with the increase of lapping time, and the energy proportion in the frequency band 8 decreased with the increase of lapping time.

The energy proportion of frequency band 7 was related to the surface of the workpiece scratched by the matrix. In contrast, the energy of frequency band 8 was connected to the scratched workpiece scratched by abrasive particles and debris. Therefore, the processing performance of FAP can be detected by the energy of frequency band 8, and the energy of frequency band 7 can detect the matrix exposure on the surface of FAP.

## Figures and Tables

**Table 1:** Process parameters of lapping experiment.

Parameter	Condition
Lapping fluid	Deionized water
Lapping pressure	26.7 kPa
Slurry flow rate Speed	50 mL/min 100 rpm

**Table 2:** Wear test parameters.

Parameter	Condition
Spindle speed	150 r/min (100 rpm)
Pressure	26.7 kPa
Wear time	1 min

**Table 3:** The corresponding frequency bands of the 8 signal nodes.

Serial Number	1	2	3	4	5	6	7	8
Node	(3,1)	(3,2)	(3,3)	(3,4)	(3,5)	(3,6)	(3,7)	(3,8)
Frequency band (Hz)	0–0.625	0.625–1.25	1.25–1.875	1.875–2.5	2.5–3.125	3.125–3.75	3.75–4.375	4.375–5

**Table 4:** Surface roughness and three-dimensional surface parameters of the FAP in each processing stage.

Lapping Time	Sa/nm	Sp/nm	Sq/nm
30 min	1294.08	7855.92	1713.72
60 min	966.01	6186.56	1372.41
90 min	940.32	5778.66	1343.55
120 min	873.51	5018.38	1251.19
150 min	830.26	4629.98	1190.13
180 min	782.09	3626.41	1100.47

**Table 5:** The surface roughness of lapping quartz glass of FAP samples at each processing stage.

Lapping Time	Sa/nm
30 min	1028.838
60 min	787.058
90 min	697.936
120 min	657.071
150 min	577.03
180 min	541.184
